# Food behaviours and eating habits among Sub-Saharan African migrant mothers of school-aged children in South Australia

**DOI:** 10.1016/j.jmh.2022.100149

**Published:** 2022-12-30

**Authors:** William Mude, Tafadzwa Nyanhanda

**Affiliations:** a School of Health, Medical and Applied Sciences, Central Queensland University, 42-52 Abbott Street & Shields Street, Cairns, QLD 4870, Australia; b School of Health, Medical and Applied Sciences, Central Queensland University, Melbourne, Australia

**Keywords:** Acculturation, Dietary changes, Eating habits, Migrant/refugee health, Traditional diets

## Abstract

Overweight, obesity and chronic conditions like diabetes, stroke and heart disease represent a significant burden to public health. Traditional foods and healthy dietary habits can reduce the risk of these conditions. Therefore, this study aimed to explore traditional food patterns and eating habits among Sub-Saharan African migrant mothers of school-aged children in South Australia. The study was a qualitative inquiry that used face-to-face interviews with 15 mothers of school-aged children in South Australia. Snowballing was used to sample participants, and data were audio-recorded, transcribed verbatim, coded, and analysed thematically. Four broad themes described participants’ food behaviours and eating habits, including maintaining traditional food patterns, changes in traditional food patterns and eating habits, concerns with food environments in Australia, and challenges with traditional food availability and access in Australia. This study found that although mothers were committed to maintaining their traditional foods, they found it increasingly difficult to continue such habits. Participants reported challenges as their children are increasingly demanding westernised foods. While some parents pushed back against such demands from their children, others felt helpless and relented. Some views showed that food environments, food systems, access, and scarcity of traditional foods in Australia influenced the participants’ food patterns and eating habits. Appropriately tailored healthy eating health promotion actions targeting school-aged children and mothers in this population need to consider their food contexts. Promoting the use of traditional foods, their preparation practices, and processing might be helpful in this community when developing healthy eating programs.

## Highlights

• Traditional foods reduce obesity and chronic diseases like diabetes and stroke.
• Traditional food practices are largely maintained but challenges exist in sourcing them.
• Food systems, supplies and environments shaped changes in food behaviours.
• Intergenerational tensions reported in food choices between mothers and their children.
• Food environments in Australia influenced children's preferences for readymade foods.

## 1 Introduction

Food culture and acculturation are important determinants of health among migrants (Batis et al., 2011). Among other factors, when people relocate from one place to another, they may experience changes in their dietary patterns and eating habits. Satia-Abouta et al. (2002) termed adopting new diets of the host country as ‘dietary acculturation’. Dietary acculturation among migrants in western countries is dynamic and multifaceted. It has been linked to adverse health outcomes among these populations. For example, adopting new diets high in sugar and dietary patterns high in fat but low in fruits and vegetables has been associated with increased overweight and obesity among immigrants (Batis et al., 2011; Satia-Abouta et al., 2002). Obesity and overweight are risk factors for cardiovascular diseases, type 2 diabetes, blood pressure, and cancer (Bendor et al., 2020; Delavari et al., 2013). Migrants from low-and-middle-income countries are particularly at risk of developing cardiovascular diseases following their migration because of changes in their food and eating habits (Jin et al., 2017; Kumanyika, 2019; Renzaho, 2016).

Scholars have generally accepted that when migrants from low-and-middle-income countries arrive in high-income countries, they are generally healthier than their host communities (McDonald and Kennedy, 2004; Renzaho, 2016). Renzaho (2016) observed that rigorous pre-migration health screening policy and traditional food patterns are important factors that contribute to health advantage among immigrants. However, McDonald and Kennedy (2004) found that as the length of time in the host countries increases, the health of migrants converges with the health status of the host communities, and this has been termed the ‘healthy immigrant effect’. Evidence suggests that the health of migrants would eventually become worse than that of the host community because of changes in eating habits and diets (Alidu and Grunfeld, 2018; Hamiel et al., 2019).

In addition to dietary acculturation, several other complex webs of factors shape dietary changes and eating habits among migrants. Available research shows that affordability, access, and knowledge of food, cultural beliefs, behavioural practices, and environmental change impact dietary changes and eating habits among migrants (Addo et al., 2019; Franzen and Smith, 2009; Mude et al., 2013; Wu and Smith, 2016). Migrants face difficulties negotiating between the beliefs of their countries of birth and their host countries, for example, selecting diets, beliefs about foods, and weight status (Addo et al., 2019; Blanchet et al., 2018). Challenges such as making decisions about diets and lack of nutritional knowledge and resettlement related stressors can also shape changes in diets, eating habits, and lifestyles (Lindberg and Stevens, 2011; Mude and Mwanri, 2016). Therefore, these factors can have implications for health outcomes in migrant families, especially when parents are not familiar with the foods in the host communities.

Understanding food behaviours and eating habits can shed light on food consumption in migrant communities so they can be supported to prevent future morbidities (Gallegos et al., 2019). In Australia, type 2 diabetes is a significant public health issue, with a prevalence of 5.3% in outer regional and remote areas (AIHW, 2020), and people from culturally and linguistically diverse backgrounds are particularly at risk of cardiovascular diseases, with the population experiencing a greater prevalence of diabetes and poorer outcomes than Australian-born people (Metro North Hospital and Health Service, 2018; TewariI and Lin, 2019). One Australian study found that immigrant children from low-and-middle-income countries had higher overweight/obesity rates across all ages (Zulfiqar et al., 2019). Mothers play important roles in shaping healthy eating in families, particularly children's food preferences and consumption patterns, and they provide a primary role as food-preparer and a gatekeeper of family food choices (Ristovski-Slijepcevic et al., 2010). Therefore, it is vitally important to understand the measures used by mothers to provide healthy foods to their families following migration. Such understanding can guide a response to prevent health risks associated with food acculturation and better support healthy eating in migrant communities.

While the health issues of dietary acculturation among migrants are well recognised, few qualitative studies have investigated the measures migrants take to provide healthy foods for their families after migration. Mothers of school-aged children play a vital role in sourcing and preparing family foods for their families and are therefore well-placed to give insights into their families’ food patterns and eating habits. This study aims to shed light on how Sub-Saharan African migrant mothers of school-aged children in South Australia describe their family food pattern and the factors that shape their families’ food behaviours and eating habits.

## 2 Methods

This study draws on the social-ecological model of health that provides a framework for understanding the multiple levels of a social system and interactions between individuals and the environment within this system (Kumar et al., 2012). According to Fielding et al. (2010), the social-ecological model emphasises the importance of the social and the physical environments that powerfully shape disease and injury patterns and our responses to them over the entire life course. There are four levels associated with the model, including intrapersonal, interpersonal, community and societal level (Dahlgren and Whitehead, 1991). The intrapersonal level relates to individual level factors that determine health outcome such as age, gender, immunity level and existing health conditions. The interpersonal level pertains to factors that determine individual health because of a person's relationship with people around them, such as friends, relatives, colleagues, and families. For example, individual may engage in certain behaviours because of peer pressure. The community level refers to factors at the community level that influence individual health outcomes example cultural beliefs, employment opportunities, health care services and many more. The societal level involves policy that influence individual health outcomes, for examples, government policy on health insurance, social services, and education. See Dahlgren and Whitehead (1991) for further information on this model.

The model has been used widely in studies that examined dietary habits and food behaviours. For example, Sogari et al. (2018) used socio-ecological model of health to explore healthy eating behaviour and dietary habits among college students in the United States of America. Amore et al. (2018) used this model to determine how perceived barriers and enablers of healthy eating among college students in Hawaii relate to the four levels (intrapersonal, interpersonal, community settings, and societal) of the model. In this paper, social-ecological model was used as a lens to analyse and interpret the study to better understand how food systems, environments, and dietary acculturation shaped food behaviours and dietary habits among Sub-Saharan African migrant mothers of school-aged children in South Australia.

The study employed a qualitative approach using face-to-face interviews with adult Sub-Saharan African migrant mothers in South Australia. Interview participants were identified and recruited through snowballing technique, a non-probability sampling of a convenience sample of initial participants to identify a hidden or hard-to-reach population (Heckathorn, 2011; Naderifar et al., 2017; Patton, 2002). The recruitment process started with the first author identifying a convenience sample of mothers of school-aged children (wave one) from South Australian urban centres. The first initial samples of participants were identified from African community activities and events held in South Australia. The first author approached the mothers of school-aged children about the study and subsequently recruited them to participate in the research.

The first author asked wave one mothers to recommend other people to join the study and provided them with flyers containing information about the study and the contact details of the researchers. The people identified by wave one respondents contacted the first author by phone or verbally to express their interest in the study, and they became wave two participants. The researcher also asked wave two participants to recommend other people (wave three participants). This recruitment process continued until no further information was forthcoming from asking the subsequent participants (Fusch and Ness, 2015; Geddes et al., 2018). Interested participants were included if they (1) were mothers of school-aged children, (2) were Sub-Saharan African, (3) lived in South Australia, and (4) volunteered to participate in the study.

Interviews were conducted in English and occurred at different locations, including community libraries, community centres and homes of the participants and lasted between 25 and 75 min. The researcher's safety was appropriately considered. The interviews explored participants’ views around traditional food patterns, food preparations and processing, eating habits, and types of foods consumed traditionally and how these have changed since migrating to Australia. All participants completed written and verbal consents before participating in the study, and each received a $30 shopping voucher card for their participation in the study. Interviews were electronically recorded and transcribed verbatim into texts by the first author.

Data analysis was informed by an interpretative approach performed by two authors, WM and TN. The two authors conducted the analysis by reading and coding the transcribed texts independently and gauging how the texts described the participants’ food behaviours and eating habits; and how these changed over time. Relevant texts that illuminated the participants’ understanding of food behaviours and eating habits were identified and coded. The changes that occurred over time were also identified and assigned codes.

**Fig. 1:**
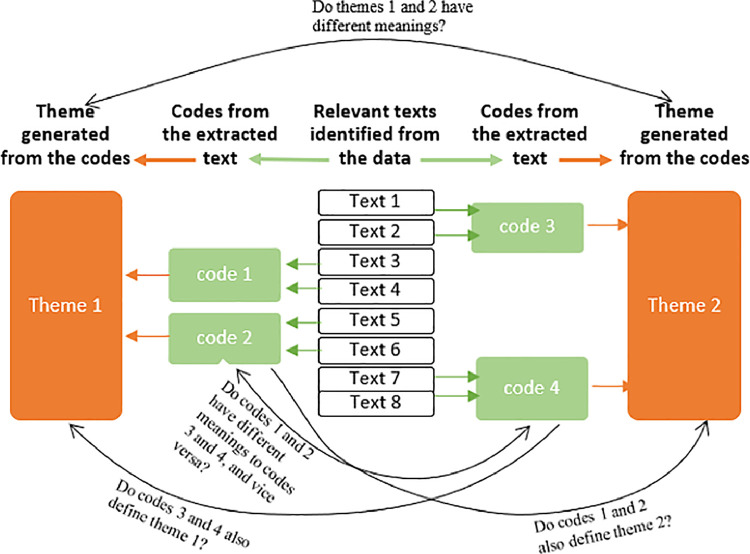
Analytic process used to code and identify themes.

The codes were categorised and combined into important themes that illuminate our understanding of food behaviours and eating habits among the participants; and how these changed over time (Maguire and Delahunt, 2017). Data analysis by coding and generating themes continued until thematic saturation was reached. Thematic saturation was achieved by ensuring that the analysis revealed no new information relating to how the participants described their food behaviours and eating habits; and how these changed over time (Lowe et al., 2018). NVivo 12 Pro software was used to manage and organise the data during the analysis. The two authors met and discussed their analysis to assess discrepancies and reach a consensus. Finally, the authors reviewed and examined the identified themes in light of the research question and the transcribed texts to determine the study's findings (Castleberry and Nolen, 2018). Fig. 1 shows the analytic steps taken to identify codes, generate themes and interrogate them to ensure they captured the information as described by the participants.

Human Research Ethics Committee from the University of Adelaide approved this study.

## 3 Results

Fifteen mothers of school-aged children participated in the study. The participants were originally from South Sudan, Sudan, Liberia, Sierra Leon, the Democratic Republic of Congo (DRC), and Somalia. They had varying lengths of stay in Australia, ranging from two to twelve years, and a majority have been living in Australia for more than ten years. Table 1 below demonstrates participant characteristics.

**Table 1:** . Participant characteristics.

Country of origin	Age range			Length of stay in Australia		
	18–24 years	25–34 years	35–49 years	< 5 years	5–10 years	>10 years
South Sudan	2	3	1	1	1	4
Sudan	1		2		1	2
Liberia		2		1		1
Sierra Leon	1					1
DRC	1					1
Somalia		2				2

The study identified four themes that broadly encompass food behaviours and eating habits described by the study participants: maintaining traditional food patterns, changes in traditional food patterns and eating habits, concerns with food environments in Australia, and challenges with traditional food availability and access in Australia.

### 3.1 Maintaining traditional food patterns

Participants reported mainly consuming foods familiar to them from their country of origin when asked to describe the types of foods they ate in Australia. Commonly consumed foods mentioned by participants included foods like spinach, cabbage, *sukuma* (Chinese kale), *mulukhiyah* (nalta jute), cassava leaves, okra, broccoli, carrots, fresh and dried meat, smoked fish, peanut butter, shea nut oils, finger millet, and chicken. In the following quote, Rose listed the common foods they ate and believed these foods played an essential role in maintaining their health. The excerpt also provides a glimpse into how Rose believed their cultural ways of cooking ‘meat in different ways’ is important for their health.

We eat sukuma, spinach, and cabbage, and I think we are healthy because of that. And although we buy meat, we cook the meat in different ways, in the ways we know from our culture. (Rose)

Betty also mentioned similar foods but added ‘smoked fish and shea nuts oil’ to the lists and how they sourced these traditional foods from Africa because of their health benefits;

“We also cook smoked fish and eat shea nuts oil, which is brought all the way directly from Africa for us here in Australia, and that is why our health is good.” (Betty)

The quotes from Rose and Betty demonstrate that participants in this study continued eating their traditional foods and cultural cooking practices in Australia. Cooking practices that alternated different foods were also commonly reported, with participants attributing their good health to such practices. Deborah captured this sentiment when she stated that “Today, you find we cook just meat by itself, another day, you find we dried it and cook it with peanut butter, and then on another day you find we cook it with okra, just like that; that's why you find up to today, we don't have any effect on us like sickness”.

In addition, many participants indicated that they still practised their traditional ways of preparing and processing foods. For example, participants mentioned they processed fish and meat by drying them to suit their preferences and removing fats. Lily revealed that she bought “fresh fish from Chinese stores and then dried the fish” because she liked “to eat fish but not the fresh ones”. Unlike Lily, Atim dried fresh meat to remove fats and used them in a specific cuisine when she noted, “We also dry meat because dried meat sometimes you can make it to your family with dried okra and does not have fat like fresh meat.”

The foods mentioned by participants in this study were mainly vegetables, cereals, poultry, and fresh or dried meat and fish. Interestingly, the commonly mentioned foods did not include any foods containing dairy and fruits.

### 3.2 Changes in traditional food patterns and eating habits

Although participants revealed they continued to eat their traditional foods, some views showed that they experienced changes in their traditional dietary practices following their resettlement in Australia. Participants observed that they started to eat foods that were not a common part of their traditional diets, for example, pizzas, bread, rice, and spaghetti. These foods demonstrate an interesting change in food patterns, from traditional staple foods to high sugar processed foods, suggesting some level of food acculturation among participants. For example, Lily and her family frequently ate out until their social worker told them that readymade foods were not good for them because they became overweight.

When we came new, me and my kids we used to go to restaurants to eat pizzas and other readymade foods. A white lady that used to help me said, “eating in the restaurant all the time is not good because I come here and see you are eating restaurant foods and becoming so fat”. Since the time she told us that, we no longer go to restaurant because when you come to new country, you don't know the system and you look like lost. (Lily)

Like Lily, Elizabeth described how she used to buy ready-cooked chicken instead of cooking for herself before realising that the foods were “not good” and “moved on buying food items from Coles, Woolworths, and Asian shops.”

Some participants recounted how they became acculturated to new diets and eating habits in Australia because of their initial resettlement experiences. Convenience and unfamiliarity of places to buy their traditional foods were noted as reasons for the change in their diets and eating habits. Deborah, for example, described how they changed their diets from eating vegetables to meat and chicken because they were not familiar with where to buy vegetables.

We started getting used to the taste of the foods here because when we arrived here, we did not know good markets selling vegetables. All that we could see was meat and chicken, which could easily be found, and we got used to it now. (Deborah).

Also, participants reported changes in food patterns and adapting cooking practices from other migrant groups in Australia. These changes in food patterns and food preparation practices were influenced by their children's demands for such foods. The accounts from Beatrice demonstrate how she adapted food preparation practices and foods that were traditionally associated with the Chinese communities.

And I started to learn to cook some of the foods which is different in Australia. Sometimes I can cook Chinese foods to eat at home because when I cook my traditional foods sometimes, they (children) don't want. He (son) just like to eat bread and rice; can eat with soup and spaghetti and chicken. Different cooking like the Chinese foods like broccoli or carrots just like fry quick and mix those things, and then they like that food to eat and I have to cook for them at home. (Beatrice)

### 3.3 Concerns with food environments in Australia

Many participants were concerned for their children's health because foods are readily available in the fridges and the supermarkets. This made mothers like Lily to ask “How can children not be fat when they eat anything they want at any time?” Atim expressed a similar sentiment relating to the availability of unhealthy food items in Australia, and the health issues children would experience if their eating habits were not managed.

We have a lot of things, we have biscuits, we have ice cream, we have all those sweet things a lot here. And the children when you don't control them, they will eat these things all the time and they will have problem with their weights. (Atim)

Additionally, there was consensus among participants that the food environments in Australia shaped their children's preferences for Australian diets. They indicated experiencing increased demands by their children for Australian foods. These increased demands have caused tensions within families. Some mothers pushed back against the demands for westernised foods by their children, like Rose, by saying that “I never buy the foods that a child needs. I will buy the foods that I know is good for the child. I will never work with the mind of a child because a child doesn't know which food is good for their health.”

However, some parents relented to the demands of their children for westernised ultra-process high sugar content foods. Lily relented to her children's demands for readymade foods with a warning about the health impacts by saying that “If the kid doesn't want, you go and bring the readymade foods and show to him or her and say this food is not good, it can cause disease; if you become sick I'm not to be blamed.” Similarly, Beatrice narrated difficulty with her children refusing to take their traditional foods to school, and she let her children choose the foods they wanted.

For school, my children don't want to take our traditional foods, they want to take the same foods Australian kids are taking to school. And that has become difficult to me, I have to take them with me to shopping so that they can go and see the foods they want. (Beatrice)

Also, participants were concerned about the food environments in Australia and stressed the importance of healthy food choices to protect their children from weight-related health risks. There was consensus among the participants that children can be at risk of overweight if not guided in their eating habits because there are abundant foods in Australia. Betty talked about selecting healthy food options, which she perceived was her parenting responsibilities to keep her children eating healthy foods.

There is a lot of foods in here (Australia). But you just need to choose which one you want. And you really need to keep your children, as a woman, to eat healthy foods. You need to know this food is not good for them. I will buy this kind of food is healthy. And as a woman I know which foods are good from my culture. (Betty)

Grace also spoke about protecting their children by giving “them healthy foods from our culture because our culture food is healthy and is good for children to eat; and is also good for their body, not like Australians foods.” Additionally, mothers of school-aged children believed their traditional foods are natural and expressed concerns that some of the foods in Australia had chemicals/additives that prolonged their shelf lives, making the foods unhealthy.

Our foods are natural, no chemical in them. Things that make people fat here (Australia) is because there is a lot of chemical in the foods. There are healthy foods here but it is not all the food because the foods we buy stays in shopping centers like Woolies or Coles for like a month or 6 weeks or more, we don't know; even may be a year, nobody knows. (Rose)

### 3.4 Challenges with traditional food availability and access in Australia

Traditional food availability and access are essential for migrants to continue their traditional food patterns. When participants were asked how they went about sourcing their traditional foods in Australia, they reported buying their traditional foods mainly from Chinese, Indian and African stores. Atim discussed how some of their traditional foods were constantly available in Asian stores, but she occasionally found some of their traditional vegetables.

Some vegetables from our culture are not here but sometimes those Asians they bring it in their stores. When you get it you will buy it, but vegetables like spinach, kudra, and cassava leaves are continuously available here in Chinese and Indian stores. (Atim)

Deborah discussed how she shopped for their traditional foods from African and Asian shops and sourced some of her vegetables from local grocery stores.

Food like greens, spinach, we just buy it from their common groceries here. But things like kudra, okra, sukuma and cassava leaves, we buy in African shops or those Asian shops. … And also finger millet, we chase it down from Indian store to make porridge. (Deborah)

However, participants expressed challenges with sourcing their traditional foods because of several reasons. Participants revealed that when they arrived in Australia, they were not familiar with the areas in the host communities. Also, they had trouble finding information about businesses that sold their traditional foods and accessing those businesses with public transportation. Faith described how she depended on the help of other community members for the location of these businesses and transportation to reach them.

We have different kinds of our traditional foods here in Australia, but we have to find where the shops are. And this is hard if you are new to a place because you don't know where the shops are and sometimes it is far. You need someone from the community to take you there. (Faith)

Elizabeth discussed the challenge she faced finding their traditional vegetables when they arrived in Australia although she now knows where to buy them “When we arrived here, we did not know good markets with greens in them. After that, we found markets for greens and knowing their locations, and now you find we started cooking these greens just like the one back home. It is now a little better, not like before.”

## 4 Discussion

This study reports on the traditional food behaviours and eating habits among Sub-Saharan African migrant mothers in South Australia. The study found that although mothers of school-aged children continued using their traditional foods and eating habits in Australia, they also reported challenges maintaining such habits. They described experiencing increased tensions in their families because of their children's inclination and demands for western foods. These influences have shaped changes in the participants' consumption of traditional foods and eating habits. Lack of information about the availability of their traditional foods and inability to source them also influenced changes in diets.

Our study found that the traditional food items identified by participants did not include any dairy and fruits. Most of the foods identified by participants consisted mainly of legumes (spinach, okra, Sukuma etc), tubers (cassava), and cereal (finger millet), which aligns with some previous studies (Codjoe et al., 2016; Renzaho and Burns, 2006). Evidence suggests that these traditional foods are parts of traditional diets and are associated with lower rates of obesity in African migrants in Australia (Renzaho et al., 2008). Also, the current study found that Sub-Saharan African migrant mothers continued to practice their traditional ways of processing foods, which often involved smoking fresh meat or fish and sun-drying vegetables. This finding is consistent with eating habits reported among African, where eating dried fish and meat is commonly practiced (Codjoe et al., 2016).

The study found that participants experienced food acculturation and changes in their eating habits in Australia. This finding supports a recent study that found a change in food patterns, from traditional staple foods to high sugar processed foods among African migrants in Australia (Addo et al., 2019). Similar experiences with diet change were also reported among Africans migrating from rural to urban areas (Cock et al., 2019). One study found that children from African migrants acculturate faster than their parents following migration, leading to intergenerational conflict (Renzaho and Mellor, 2010), even on issues such as food choices and eating habits within families. Our study highlights this, with mothers describing how their children prefer western foods instead of traditional foods. An interesting finding from this study was that change in the types of foods consumed did not only occur in Western foods but also by adapting foods from other migrant communities in Australia. For example, participants adapted Chinese cooking styles in their food preparation practices in Australia.

However, our finding differed from Blanchet et al. (2018) study, which reported spiralling dietary changes among black (Caribbean and Sub-Saharan African) immigrant families in Canada. Our finding contradicted this result and found that many Sub-Saharan African migrant mothers in South Australia continued to eat their traditional foods and adopted some foods from the host and migrant communities. The current paper corroborates Renzaho and Burns's (2006) study, which found that Sub-Saharan Africans in Australia have maintained their traditional diets. The observed contradiction between this paper and the study by Blanchet et al. (2018) could be because of differences in food environments and systems in these locations that determine food acculturation (Handley et al., 2013).

Nevertheless, our study found that participants faced challenges with sourcing their traditional foods. A previous study showed that although traditional foods are available, they are often too expensive and not sustainable to maintain as regular diets because of the low socioeconomic status of many migrant families (Renzaho and Burns, 2006). Such a prohibitive factor could facilitate future dietary acculturation among the participants, influencing dietary-related health outcomes, such as obesity and overweight.

### 4.1 Policy recommendations

The findings from this study show some areas where public health policy and health promotion interventions should be directed. The study revealed how food acculturation, food systems and environments in South Australia shaped food behaviours and habits among the participants. This finding underscores the need for a multi-faceted healthy eating approach that considers food systems and supplies as part of its design. Traditional and cultural food production and processing practices migrants bring to Australia should be promoted and supported. For example, by engaging migrants in urban and community gardens to maintain their traditional and cultural ways of sourcing foods.

Additionally, policy actions designed to sustain healthy food systems and supplies should consider the inclusion of traditional and cultural food consumed among migrant populations in such systems and supply chains. At a government level, effort should be directed at reducing taxes for traditionally produced foods because of their health benefits to make their production and supply an easier and a cheaper option. Alternatively, the production and supply of traditionally produced foods should be subsidised. The funds for such subsidy could come from the money that would have gone to treat and manage chronic conditions linked to eating ultra-processed energy dense foods.

Given the tension in food choices between children and their mothers revealed in this study, urgent action is needed to promote the health benefits of eating traditional food among this population. In addition, health promotion activities and policies should support migrant mothers of school-aged children and their families by engaging them in culturally appropriate healthy eating programs. For example, health literacy activities that are designed to promote healthy foods and their benefits to individual and family health outcomes. Working with schools in neighbourhoods with high proportion of migrant children to improve school-aged children's awareness about healthy eating.

### 4.2 Limitations and strengths

A few limitations are that data were collected in English and recruitment was done among mothers in major South Australian urban centres. Conducting interviews in participants’ first language could have improved clarity and an in-depth understanding of this population's food behaviours and eating habits. Moreover, the focus of recruiting participants from major urban areas might have missed potential Sub-Saharan African migrant mothers in smaller towns in South Australia. As a result, while the knowledge gained from this study is valuable, the finding cannot be generalised for all the mothers of school-aged children in this community.

This study's strengths lie in its target participants and the diversities of the participants. Interviews African migrant mothers of school-aged children in this study facilitated the collection of information for a nuanced understanding of their dietary habits and food behaviour. This is critical given the lead role mothers take in the families in sourcing, processing, and preparing family meals. Inclusion of mothers of school-aged children in this study provided the participants with the opportunity to talk about the issues that matter most to them relating to food behaviours and eating habits. This opened a window of opportunity to understand their beliefs, values, and assumptions about healthy eating. Furthermore, the participants in this study were from different cultures and countries of origin. The consistent description of food behaviours and habits among the participants demonstrates the importance of these issues across cultures and countries of origin, strengthening the findings.

## 5 Conclusion

This study explored traditional food behaviours and eating habits among Sub-Saharan African migrant mothers of school-aged children in South Australia. The study uncovered areas in which healthy eating policy and interventions could be targeted to prevent health issues such as overweight, obesity and chronic conditions like diabetes, stroke, and heart disease in this population. This study found that Sub-Saharan African migrant mothers of school-aged children find it increasingly challenging to provide traditional foods to their children, highlighting the urgent need for a rethink about food systems and supply and working with parents to support their children. Food acculturation, food systems and environments in South Australia presented challenges to maintaining the consumption of traditional foods and eating habits. The finding can inform health promotion activities and policy to better support mothers of school-aged children and their families in this community. Appropriately tailored healthy eating health promotion actions targeting school-aged children and mothers in this population need to consider their food contexts. Promoting traditional foods and eating habits might be helpful in this community when developing healthy eating programs. Public health food policy must also consider systems and supply chains of traditional foods to make healthy eating an easier and cheaper option. Further population-level studies are required to corroborate the findings reported in this study.

## Funding

This study did not receive funding and was part of the first author's graduate research work.

## Declaration of Competing Interest

The authors declare that they have no known competing financial interests or personal relationships that could have appeared to influence the work reported in this paper.
